# An Incidental Finding of Gain of a Diminished Chromosome 12 Centromere in an Individual with Lymphocytosis: A Case Report and Clinical Implications in Cytogenetic Testing

**DOI:** 10.3390/diagnostics15050618

**Published:** 2025-03-04

**Authors:** Changqing Xia, Jeffrey J. Cannatella, Scott C. Smith, Pamela A. Althof, Haley Koerselman, Thomas Hempel, Erin E. Jaworski, Lisa M. Winkler, Joanna R. Spaulding, Diane Pickering, Joseph D. Khoury, Zhenya Tang

**Affiliations:** 1 Department of Pathology, Microbiology and Immunology, University of Nebraska Medical Center, Omaha, NE 68198, USA; 2 Department of Pathology, Upstate Medical University, Syracuse, NY 13210, USA

**Keywords:** centromere 12, centromere 16, rearrangement, fluorescence in situ hybridization (FISH), chromosomal analysis, incidental finding

## Abstract

**Background:** Fluorescence in situ hybridization (FISH) testing against chromosome 12 centromere (CEN12) is routinely included in the work-up of patients with suspected chronic lymphocytic leukemia (CLL) or monoclonal B-cell lymphocytosis (MBL). However, incidental findings can occur and be challenging. **Methods**: Interphase and metaphase FISH analyses with various probes, including CEN12 probes from different vendors, and conventional cytogenetics were applied. **Results**: A CLL FISH panel was performed at the clinician’s request on a peripheral blood specimen from a 55-year-old female with fluctuating leukocytosis and lymphocytosis for over six years. An additional diminished CEN12 FISH signal was observed in approximately 70% of the nucleated cells analyzed. Concurrent flow cytometry excluded a diagnosis of CLL or MBL, and karyotyping exhibited a normal female karyotype. Further studies excluded potential cross-hybridization due to limited specificity of the CEN12 probes and revealed the location of the additional diminished CEN12 signal on the centromere of one chromosome 16 homolog (CEN16), without other material from the short arm (12p) or long arm (12q) of chromosome 12 being involved. **Conclusions**: This is the first case with an “uncertain” trisomy 12 status, presenting a challenge to clinical cytogenetic diagnosis. Although the mechanism for this mosaic “partial trisomy 12” and its clinical impact remain unknown, this case highlights the importance of further investigation using orthogonal methods to clarify incidental findings during diagnostic practice.

## 1. Introduction

The gain of an additional copy of chromosome 12, known as trisomy 12 (+12), is a recurrent chromosomal aberration found in patients with chronic lymphocytic leukemia (CLL)/small lymphocytic lymphoma (SLL) (referred to as CLL hereafter) [1,2,3,4]. The prevalence of +12 in CLL is 10% to 25%, making it the second most frequent chromosomal aberration, next to the deletion of 13q (del(13q)) and followed by the deletion of 11q (del(11q)) and the deletion of 17p (del(17p)) [1,2]. These recurrent chromosomal aberrations are collectively termed “CLL-associated”.

Trisomy 12 positive CLL cells, or “+12 CLL” cells, often present unique characteristics, such as atypical morphology (e.g., unusual cleaved or lympho-plasmacytoid appearance under a microscope), increased proliferation rate, a higher expression of CD38, unmutated immunoglobulin heavy chain variable region (*IGHV*) genes, positive zeta chain-associated protein kinase 70 (ZAP70) expression, Notch Receptor 1 (*NOTCH1*) gene mutation, a high risk of Richter transformation, and so on [5]. The presence of +12 is considered a marker for an intermediate prognosis in CLL [1,2,3,4].

The CLL-associated chromosomal aberrations have been observed in individuals with monoclonal B-cell lymphocytosis (MBL), a non-malignant hematologic condition characterized by an expansion of clonal B cells in the blood without lymphadenopathy, organomegaly, or symptoms [6,7,8]. According to the World Health Organization (WHO) Classification of Hematolymphoid Tumors [9], MBL is classified into three subtypes: low-count MBL (LC-MBL) with a clonal B-cell count < 0.5 × 10^9^/L and without CLL-like phenotype; CLL-like MBL with a clonal B-cell count of 0.5 to 5 × 10^9^/L and with CLL-like phenotype; and non-CLL-like MBL with a clonal B-cell count of 0.5 to 5 × 10^9^/L and with non-CLL-like phenotype. Most non-CLL-like MBL cases can present features of marginal zone lymphoma (MZL) [9].

In a recent study of 4031 individual specimens analyzed with fluorescent in situ hybridization (FISH) and single nucleotide polymorphism (SNP) array assays, including 2971 without MBL (control group), 728 with LC-MBL, and 332 with HC-MBL, Sekar et al. [10] demonstrated that the detection rate of CLL-associated chromosomal aberrations, also including the deletion of 6q (del(6q)), is significantly higher in the HC-MBL (52.1%) and LC-MBL (1.1%) groups than in the control group without MBL (0.13%). Therefore, a CLL FISH panel targeting the four recurrent chromosomal abnormalities mentioned above is commonly requested and performed as part of the standard workup for all cases with a suspected diagnosis of CLL or MBL. Since chromosomal aberrations involving chromosome 12 mostly present as a gain of a whole chromosome 12 or a complete trisomy 12 (+12) in both CLL and MBL, a centromeric probe for chromosome 12 is included in a CLL FISH panel, along with locus-specific probes for del(11q), del(13q), and del(17p) aberrations [11,12,13,14,15].

FISH probes employed for clinical diagnostics usually achieve a specificity of 100% and a sensitivity of >95%, as stated in the American College of Medical Genetics and Genomics (ACMG) technical standards and guidelines for FISH testing [16]. A specific FISH test needs be comprehensively validated before its clinical use, and proficiency testing is periodically implemented to ensure its performance [16,17]. Therefore, FISH test results are generally considered as being definite and straightforward. However, incidental findings of FISH tests can occur, and interpreting them can be challenging.

We report here an incidental finding of an additional, diminished centromere 12 signal in the peripheral blood of an individual with a fluctuating history of leukocytosis/lymphocytosis, suspected of having CLL or MBL. Further characterization demonstrated that the additional, diminished centromere 12 signal is located on the centromeric region of a chromosome 16. We also confirmed that no proximal regions on either of the 12p or 12q arms are located on chromosome 16. To the best of our knowledge, this is the first case with an “uncertain” trisomy 12 status. Although the clinical significance of this phenomenon is yet unknown, this case study highlights a rare event of centromere rearrangement and its clinical implications for cytogenetic testing.

## 2. Case Report

A 55-year-old female presented with a history of intermittent slight elevation of white blood cells (WBCs) and lymphocytosis for over 6 years. Her lymphocytosis was incidentally identified in a routine blood test, but her WBCs and lymphocytes remained relatively consistent above the normal range. The test results of WBC counts, lymphocytes percentages, and absolute lymphocyte counts at 12 timepoints over 6 years are listed in Table 1. The patient had a history of Type II diabetes for approximately 12 years, currently controlled with tirzepatide and semaglutide. She also had surgical histories of hysterectomy at age 36 because of menorrhagia, cholecystectomy, and appendectomy 15 years prior. Additionally, she had elevated cholesterol and hypertension, both under medical treatment. The patient was married with three sons, and she had no remarkable family history or social history.

**Figure 1:**
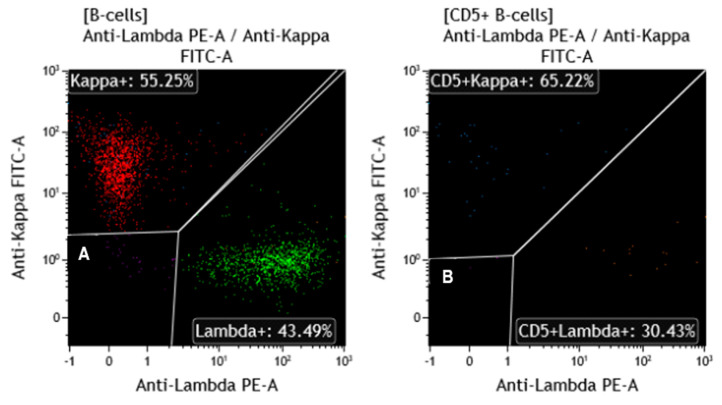
Total B-cells (**A**) and CD5-positive B-cells (**B**) show no evidence of light chain restriction by flow cytometry.

**Figure 2:**
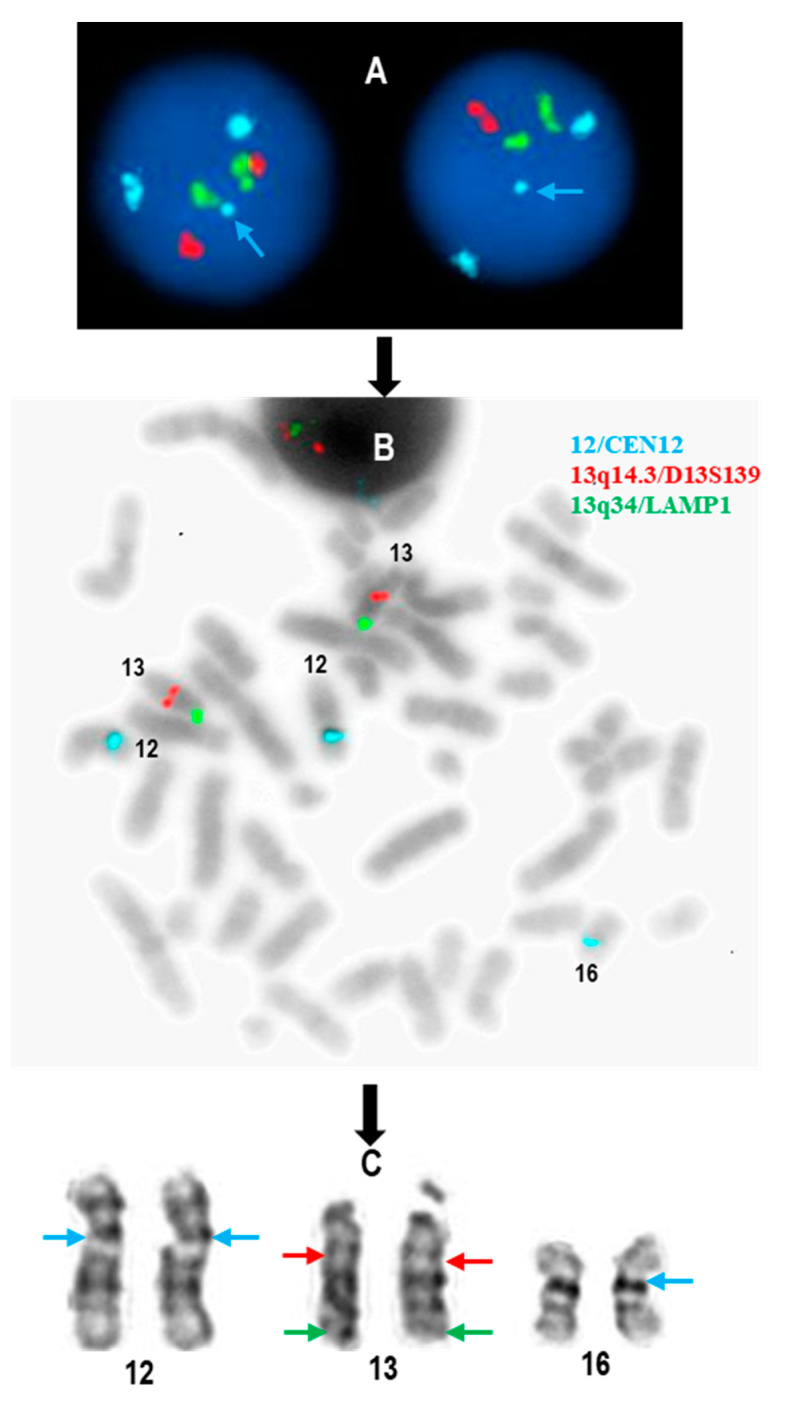
Initial FISH and chromosomal analysis findings in this individual. (**A**) Two abnormal interphase FISH (12/*CEN12*, 13q14.3/*D13S139*, and 13q34/*LAMP1*) images indicating gain of a diminished *CEN12* signal (aqua) (see arrows). (**B**) An inverted metaphase FISH image shows that the additional, diminished *CEN12* signal is located on a chromosome 16. (**C**) Illustration of FISH signals on the morphologically normal chromosomes 12, 13, and 16 respectively.

During her most recent visit, flow cytometry analysis of her peripheral blood did not detect a monotypic B-Cell population (Figure 1), showing polyclonal B cells in both the whole B cell population (Figure 1A) and CD5+ B cell population (Figure 1B). Concurrently, a CLL FISH panel using probe sets of 11q22.3/*ATM*, 17p13.1/*TP53* (Leica Biosystems, Deer Park, IL), 6q/*MYB*, 13q14.3/*D13S319*, 13q14.3/*LAMP1*, *IGH* breakapart (BAP), t(11;14)/*IGH*::*CCND1* XT (Abbott Molecular, Abbott Park, IL, USA), and *CEN12* (Copy Control 12 Aqua, Biocare Medical, Pacheco, CA, USA) was performed. Three *CEN12* signals were observed in 141 out of 200 (70.5%) interphase cells analyzed (Figure 2A), while the other probes included in the same CLL FISH panel showed normal results. One *CEN12* signal was apparently weaker compared to the other two *CEN12* signals (Figure 2A), suggesting a diminished signal. Concurrent chromosomal analysis demonstrated a morphologically normal female karyotype (46, XX [20]). Further metaphase FISH studies indicated that the diminished aqua signal is likely located on one of the chromosome 16 homologs (Figure 2B,C).

**Figure 3:**
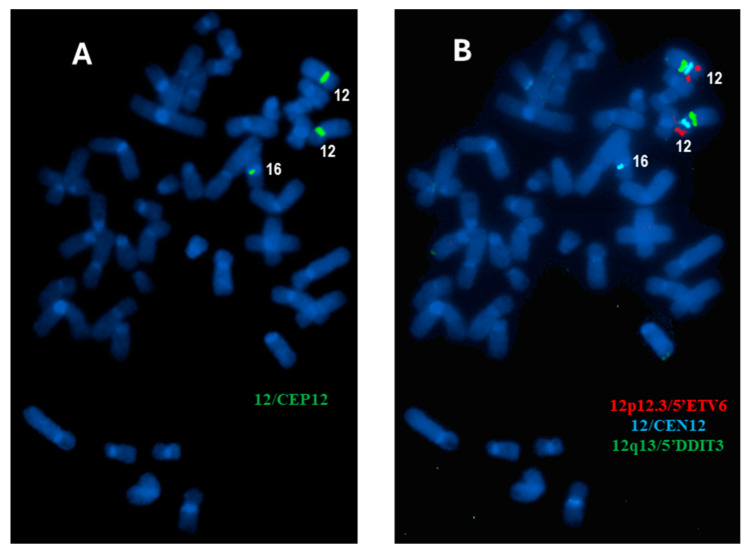
Additional FISH studies in this case. (**A**) A CEP12 probe from a different manufacturer with different fluorescence dye (green) labeling has been applied. Same results as that in the initial analysis have been obtained. The metaphase CEP12 image demonstrated the presence of a diminished CEP12 signal (green) on a chromosome 16. (**B**) A probe set targeting various portions of chromosome 12 (*12p12.3/5′ETV6*, *12/CEN12*, and *12q13/5′DDIT3*) has been applied to the same slide (after destain). The same metaphase exhibited in (**A**) was captured, indicating that likely only a small portion of centromere 12 is located on one chromosome 16, while both chromosomes 12 are normal.

To exclude the possibility of the diminished CEN12 signal being caused by potential cross-hybridization due to limited specificity of CEN12 probe from one manufacturer, a second centromere 12 probe from a different manufacturer labeled with different fluorochrome (Vysis CEP12 (D12Z3) SpectrumGreen Probe, Abbott Molecular, Abbott Park, IL, USA) was used to repeat the centromere 12 FISH. The same result of an additional, diminished centromere 12 signals (CEP12/Green) was obtained in most cells analyzed, and with the additional diminished CEP12 signal located on one of the chromosome 16 homologs (Figure 3A). The same slide was destained and re-hybridized with a probe set targeting 12p (Vysis LSI *ETV6* (*TEL*) SpectrumOrange, Abbott Molecular, Abbott Park, IL, USA), CEN12 (Copy Control 12 Aqua, Biocare Medical, Pacheco, CA, USA) and 12q (Vysis LSI *DDIT3* (*Tel*) SpectrumGreen Probe, Abbott Molecular, Abbott Park, IL, USA). The results demonstrated that only a part of centromere 12 (diminished signal) is located on the centromere region of one chromosome 16, while both *5′ETV6* and *5′DDIT3* were retained on both chromosomes 12 (Figure 3B), indicating gain of a diminished centromere 12 signal by FISH testing, which is cryptic by chromosomal analysis. Due to insufficient material obtained from this cell culture, certain planned tests, such as CEN16 FISH and whole chromosome painting (wcp) 12 and 16, cannot be performed. Since no clonality of lymphocytes was detected, a hematologic malignancy has been ruled out. The patient is currently routinely receiving follow-up in clinics.

## 3. Discussion

Centromere probes are commonly used in FISH testing to detect numerical alterations of the targeted chromosome or as a control for other targeted genes or regions located at the same chromosome [17,18,19]. For example, FISH analysis of the human epidermal growth factor receptor 2 (*HER2)* gene often utilizes a control probe against the centromeres of chromosomes 17 (CEN17) to determine whether *HER2* is amplified based on the *HER2*/CEN17 ratio [20,21,22,23].

As early as the 1990s, the centromere probes for sex chromosomes (X and Y) and chromosome 18 (CEN18), along with locus-specific probes for 13q14 and 21q22, were applied as an aneuploidy FISH panel for the rapid detection of aneuploidy status in both prenatal and postnatal diagnosis [24,25,26,27,28]. Although non-invasive prenatal screening (NIPS) has replaced the aneuploidy FISH panel in the prenatal diagnosis of aneuploidy status [29,30,31], the aneuploidy FISH panel is still recommended for confirmation or exclusion of positive or inclusive findings obtained by NIPS [28,32]. As mentioned previously, the CEN12 probe is routinely included in the FISH panel for CLL and/or MBL work-up [3,4,10]. All these are based on the fact that each non-acrocentric human chromosome possesses a chromosome-specific family of alpha satellites defined by a unique higher-order repeat (HOR) unit of each centromere. This unique HOR unit is usually considered as the identity of the centromere and can be detected with specific centromeric FISH probes through complimentary DNA hybridization [33,34,35]. In contrast, the acrocentric chromosomes (such as 13, 14, 21, and 22) usually share large homologous regions in their short arm, and cross-hybridization among these acrocentric chromosomes has been observed when alpha satellite DNA probes are used [36,37]. Therefore, alpha satellite DNA FISH probes for these acrocentric chromosomes are rarely used clinically.

Recent studies using long read sequencing technology have completely sequenced and assembled all centromeres of the human genome and demonstrated a higher frequency of single nucleotide variation (over 4-fold) and size variation (approximately 3-fold) than previously reported. This indicates rapid evolution in the centromeres of the human genome [38,39]. Due to the tandem repeats of alpha satellites at the centromeres, non-allelic homologous recombination can occur during the process of cell division and propagation, potentially leading to a segment of a centromere from one chromosome appearing on the centromeric region of another chromosome [40]. Additionally, transposable elements (TE) enriched at the centromere region can change their position, moving from one location to another, resulting in centromere transposition [41,42].

Although many other factors help stabilize the centromeres to prevent them from frequent migration, the transposition of centromeres can occur, potentially causing genetic problems and difficulties in genetic diagnosis [43,44,45]. For example, in a case reported by Giannuzzi et al. [46], the presence of three signals of CEN18 in all analyzed cells (150/150, 100%) and three signals of 21q22 in 24% (29/121) of analyzed cells was detected in an amniotic fluid specimen at 15 weeks’ gestation of a 35-year old woman for her sixth pregnancy. Her previous pregnancies resulted in two healthy children, one miscarriage of unknown reason, and two pregnancies terminated due to fetal trisomy 21. Concurrent chromosomal analysis on the cultured amniocytes confirmed the mosaic trisomy 21 (19%, 12/62), but all karyotyped cells exhibit two morphologically normal chromosomes 18. FISH analysis on the cultured amniocytes showed three copies of CEN18 signals in all cells (100%), and metaphase FISH indicated that one of the three CEN18 signals was located on chromosome 15q26. Concurrent chromosomal microarray on the unculture amniocytes detected a mosaic trisomy 21 (13%) only. Parental FISH studies showed that the insertion of a portion of CEN18 into 15q26 was de novo. Her sixth pregnancy continued unremarkably, and a healthy male baby was born at term without any abnormal phenotypes observed. The postnatal analysis of the newborn’s peripheral blood showed the same findings as the prenatal amniotic fluid specimen. Further investigation confirmed that the insertion occurred at chr15:92359068 and chr15:92361920 (GRCh38), replacing a 2852-bp segment at 15q26. The insert is approximately 10 Mb away from the ancestral centromere of the affected chromosome 15. The investigators demonstrated that the insertion in this case has no functional elements, which aligns with the absence of clinical features in the proband (his trisomy 21 mosaicism also appears asymptomatic).

Although potential cross-hybridizations between centromeric FISH probes of chromosomes 1 and 5, 1 and 19, and 5 and 19 have been reported [34], a potential cross-hybridization between centromeric FISH probes of chromosomes 12 and 16 has not been reported to date. Therefore, the additional, diminished centromere 12 signal located on one centromere 16 in our case is very unlikely to be caused by cross-hybridization, which is also supported by our FISH tests performed using CEN12 probes from different manufacturers. This may occur through non-allelic homologous recombination between the centromeres of chromosomes 12 and 16 [40], or through a transposon movement of TEs at the chromosome 12 centromere and random relocation to the chromosome 16 centromeric region [41]. The latter may better explain the fact that only a small portion of centromere 12 (a diminished signal) is on chromosome 16. Approximately 70% of cells analyzed by FISH exhibit this incidental finding, indicating a mosaic status in our case. It remains unknown whether this is a constitutional event or an acquired change, possibly even age-related. As the B-cells were immunophenotypically unremarkable, a diagnosis of MBL or CLL has been excluded. Therefore, this incidental finding likely has no clinical impact on this individual. The dynamical monitoring of the clonal size of the cell population exhibiting this incidental finding by centromere 12 FISH testing is warranted.

Translocations between chromosomes 12 and 16, or t(12; 16) aberrations, have been reported in hematological malignancies. Notably, these include fusions between the ETS variant transcription factor 6 (*ETV6*) and CREB binding protein (*CREBBP*) genes (*ETV6::CREBBP*) [47], as well as between zinc finger protein 384 (*ZNF384*) and *CREBBP* genes (*ZNF384::CREBBP*) [48,49], both through t(12; 16) (p13; p13) aberrations, which have been observed in acute lymphoblastic leukemia. Interestingly, t(12; 16) aberrations are even more common in solid tumors. For example, the t(12; 16) (q13; p11) aberration, resulting in the fusion of FUS RNA binding protein (*FUS*) and DNA damage-inducible transcript 3 (*DDIT3*) genes (*FUS::DDIT3*), has been observed in approximately 95% of myxoid liposarcoma cases [50,51]. However, to the best of our knowledge, a translocation involving centromeres of chromosomes 12 and 16 has not been reported in the literature.

In conclusion, this case study highlights a rare event of centromeric rearrangement through an unknown mechanism and its implications for clinical cytogenetic diagnosis; although, the clinical significance remains unknown. It also demonstrates that solely relying on one test result can mislead the diagnosis and clinical management. Once an incidental finding is observed, further investigation using orthogonal methods is warranted to clarify the incidental finding.

## Figures and Tables

**Table 1:** Dynamic changes of peripheral blood WBCs, lymphocyte percentages, and absolute lymphocyte counts in this individual over 6 years.

Test Results (Normal Range)	Time (Month)											
	0 *	13	26	29	38	55	57	62	66	67	69	73
WBC (normal range 4.0–11.0 × 10^3^/μL)	12.7 ^	8.4	10.2 ^	10.1	10.1	11.8 ^	13.1 ^	13.1 ^	14.2 ^	14.9 ^	10.6	11.8 ^
Lymphocytes %	43	44	38	43	36	42	38	35	39	45	36	34
Lymphocytes absolute (normal range 0.7–3.9 × 10^3^/μL)	5.5 ^	3.7	3.9	4.3 ^	3.6	5 ^	5 ^	4.6 ^	5.5 ^	6.7 ^	3.8	4 ^

* Initial abnormal finding; ^ above normal range.

## Data Availability

The original contributions presented in this study are included in the article. Further inquiries can be directed to the corresponding author(s).

## Abbreviations

ACMG	American College of Medical Genetics and Genomics
ATM	ataxia-telangiectasia mutated gene
BAP	breakapart
CCND1	cyclin D1 gene
CEN	centromere
CLL	chronic lymphocytic leukemia
CREBBP	CREB binding protein
DDIT3	DNA damage-inducible transcript 3
ETV6	ETS variant transcription factor 6
GRCh38	genome reference consortium human build 38
HC-MBL	high-count monoclonal B-cell lymphocytosis
HER2	epidermal growth factor receptor 2
IGH	immunoglobulin heavy chain gene
IGHV	immunoglobulin heavy chain variable region
LAMP1	lysosomal-associated membrane protein 1 gene
LC-MBL	low-count monoclonal B-cell lymphocytosis
MBL	monoclonal B-cell lymphocytosis
MYB	myeloblastosis gene
MZL	marginal zone lymphoma
NIPS	non-invasive prenatal screening
NOTCH1	Notch Receptor 1 gene
SNP	single nucleotide polymorphism
SLL	small lymphocytic lymphoma (SLL)
TE	transposable element
TP53	tumor protein 53 gene
WBC	white blood cell
ZAP70	zeta-chain-associated protein kinase 70
ZNF384	zinc finger protein 384
